# Invasive lobular carcinoma of the breast; clinicopathologic profile and response to neoadjuvant chemotherapy over a 15-year period

**DOI:** 10.1016/j.breast.2024.103739

**Published:** 2024-05-01

**Authors:** N.P. Quirke, C. Cullinane, M.A. Turk, N. Shafique, D. Evoy, J. Geraghty, D. McCartan, C. Quinn, J.M. Walshe, E. McDermott, C. Rutherford, R.S. Prichard

**Affiliations:** a UCD School of Medicine, University College Dublin, D04 V1W8, Dublin, Ireland; b Department of Breast and Endocrine Surgery, St. Vincent's University Hospital, Dublin, Ireland; c Royal College of Surgeons in Ireland, Dublin, Ireland; d Department of Pathology, St. Vincent's University Hospital, Dublin, Ireland; e Department of Oncology, St. Vincent's University Hospital, Dublin, Ireland

**Keywords:** Breast, Lobular, Cancer, Neoadjuvant chemotherapy

## Abstract

**Introduction:**

Invasive lobular carcinoma (ILC) accounts for 5–15% of invasive breast cancers. Typical ILC is oestrogen receptor (ER) positive and human epidermal growth factor receptor 2 (HER2) negative. Atypical biomarker profiles (ER- and HER2+, ER+ and HER2+ or triple negative) appear to differ from typical ILCs. This study compared subtypes of ILC in terms of clinical and pathological parameters, and response to neoadjuvant chemotherapy (NACT) according to biomarker profile.

**Methods:**

All patients with ILC treated in a single centre from January 2005 to December 2020 were identified from a prospectively maintained database. Clinicopathologic and outcome data was collected and analysed according to tumour biomarker profile.

**Results:**

A total of 582 patients with ILC were treated. Typical ILC was observed in 89.2% (n = 519) and atypical in 10.8% (n = 63). Atypical ILCs were of a higher grade (35% grade 3 vs 9.6% grade 3, p < 0.001).

A larger proportion of atypical ILC received NACT (31.7% vs 6.9% p < 0.001). Atypical ILCs showed a greater response to NACT (mean RCB (Residual Cancer Burden Score) 2.46 vs mean RCB 3.41, p = 0.0365), and higher pathological complete response rates (15% vs 0% p = 0.017). Despite this, overall 5-year disease-free survival (DFS) was higher in patients with typical ILC (91% vs 83%, p = 0.001).

**Conclusions:**

Atypical ILCs have distinct characteristics. They are more frequently of a higher grade and demonstrate a superior response to NACT. Despite the latter, atypical ILCs have a worse 5-year DFS which should be taken into consideration in terms of prognostication and may assist patient selection for NACT.

## Highlights

• ILC is a unique clinical entity and should be considered separately to IBS-NST for research and treatment purposes.
• Biomarker profile influences ILC disease behaviour and may have an effect on response to neoadjuvant chemotherapy.
• Tumour size and lymph node status are independent predictors of recurrence free survival in ILC.

## 1 Introduction

Invasive lobular carcinoma (ILC) is the second most common subtype of breast cancer, accounting for 5–15% of all invasive breast cancers [1]. Its unique clinical and pathological features distinguish it from the more common invasive breast carcinoma no special type (IBC-NST) breast carcinoma. ILC shows a characteristic dyscohesive and infiltrative growth pattern, most commonly attributed to the loss of the cell adhesion molecule, E-cadherin [2]. The growth pattern seen in ILC may render it mammographically occult and difficult to diagnose clinically [3]. These are likely contributory factors to the more advanced T stage at presentation that may be associated with ILC. ILC also shows a tendency towards multifocality and bilaterality as compared to other forms of invasive breast cancer [4].

Clinical breast cancer research does not always distinguish between IBC-NST and ILC. However, these are distinct entities, both in terms of pathological profile and response to treatment [5,6]. Regarding oncological outcomes, reported survival comparisons between IBC-NST and ILC vary, sometimes with conflicting evidence. Pestalozzi et al. demonstrated that ILC has improved disease-free survival benefits in the early years after diagnosis compared to IBC-NST, with this alternating in the longer term, with worse overall and disease-free survival observed in patients with ILC [7,8]. In contrast, Chamalidou et al. recently reported that overall survival is similar in the two groups [9].

The typical biomarker profile of ILC is oestrogen receptor (ER) positive and human epidermal growth factor receptor 2 (HER2) negative, with adjuvant endocrine therapy the mainstay of systemic treatment. This profile is observed in approximately 93% of cases of ILC [8,10]. A small proportion of ILCs are HER2 positive or triple negative, particularly in the less common histological subtypes [11]. While studies have characterised the various histological subtypes of ILC, a distinct clinical behaviour associated with the biomarker subgroups of ILC, determined using immunohistochemistry (IHC) and *in situ* hybridisation techniques as indicated, has not been clearly demonstrated [10].

In general, ILCs respond poorly to neoadjuvant chemotherapy (NACT), with current practice recommending this form of treatment to facilitate surgery for locally advanced ILC tumours [12,13]. Pathological complete response rates (pCR) for ILC are reportedly lower than for IBC-NST [14]. The aim of this study was to examine the biomarker subgroups of ILC and to compare the clinical and pathological parameters of typical ILC (ER+, HER2-) to those of atypical ILC, including response rates to NACT.

## 2 Methods

A retrospective review was performed on a prospectively maintained database of all patients treated for ILC at a single tertiary referral centre over a fifteen-year period, from January 2005 to December 2020. Patients diagnosed through both symptomatic clinics and Ireland's national breast screening programme (BreastCheck) were included.

Patients were excluded from the study if they had metastatic disease at diagnosis, declined treatment, exhibited a mixed ductal/lobular histological phenotype or did not have a tumour biomarker profile documented.

Clinical and pathological data including age at presentation, tumour size, tumour grade, tumour biomarker profile, presence of lymphovascular invasion (LVI) and nodal status were recorded from hospital pathology databases. Patient follow-up, incorporating recurrence-free survival (RFS) was obtained using the hospital radiology database and clinic letters, taking a patient's most recent surveillance mammogram or other relevant investigations to assess for evidence of loco-regional or distant recurrence. Recurrence-free survival was calculated from the time of biopsy confirmed diagnosis of primary breast cancer to the date of the first recurrence event.

ILC was diagnosed and categorised histologically in accordance with accepted definitions [15]. Loss of E-cadherin on immunohistochemistry supported tumour designation as ILC but this was not a requirement for diagnosis, as retention of E-cadherin is reported in up to 15% of ILC tumours [16]. Tumours were graded according to the Nottingham grading system [17]. Lymphovascular invasion (LVI) was reported as either absent, possible or present in the tissue surrounding the tumour. Tumour size, invasive and whole, was confirmed microscopically in accordance with standard protocol [18].

Typical ILC was defined as hormone receptor (HR) (ER ± progesterone (PR)) positive and HER2 negative. ILC was categorised as atypical if the biomarker profile was ER positive and HER2 positive, ER negative and HER2 positive, or triple negative (ER/PR/HER2 negative).

Tumours were considered to be ER and/or PR positive when >1% of cells showed positive staining. PR staining was not performed routinely at our institute until 2019. HER2 status was assessed by IHC and by Fluorescent in Situ Hybridization (FISH) in tumours with a 2+ IHC result. HER2 positivity was defined as either 3+ with IHC and/or amplified with FISH.

Patients received NACT according to standard guidelines. Patients with HER2 positive tumours received HER2 blockade (trastuzumab) concomitantly with NACT. From July 2020 patients received dual HER2 blockade with pertuzumab and trastuzumab in line with national guidelines. In patients with more than one tumour with different biomarker profiles, the profile that directed adjuvant treatment was recorded.

In patients who received NACT, pathological response was assessed using the Residual Cancer Burden score (RCB) [19]. RCB was included in the majority of histopathology reports from 2018. Where this had not been included in the histopathological report and sufficient pathological data were available, RCB was calculated retrospectively using the MD Anderson Cancer Centre's online calculator [20]. pCR is defined as no residual invasive disease (or lymphovascular invasion) in either the breast or lymph nodes (ypT0N0 or ypTisN0).

Patient demographics and pathological details were evaluated using descriptive statistics assessed using the Fisher exact test for categorical variables and the chi-squared test for continuous variables. The primary study endpoint was recurrence-free survival. Kaplan-Meier plots were generated according to selected tumour characteristics log-rank testing to measure survival differences between groups. To assess the independent prognostic significance of tumour characteristics univariate cox-proportional hazards models were used and performed on the entire cohort. Subsequently, multivariate cox proportional hazards model using variables with p < 0.1 in univariate analysis was performed. Statistical analysis were performed using R Studio (“Ghost Orchid Release” for Mac OS 2021.09.1) and SPSS version 26. The study was powered using G power analysis software to show a 5% difference in recurrence-free survival rates with effect size of 0.80 and alpha error of 0.05.

## 3 Results

### 3.1 Patient cohort characteristics

A total of 582 patients were treated for ILC and met the inclusion criteria during the study period. All 582 patients were female. Typical ILC (HR+, HER2-) was the predominant subgroup, accounting for 519 (89.2%) tumours. A total of 553 (95%) of ILCs were HR positive. Eight (1.4%) tumours were HR negative and HER2 positive, and 21 (3.6%) were triple negative (Table 1).

**Table 1:** Tumour biology characteristics.

Receptor Status	n =	% of total
ER +, HER 2 – (Typical)	519	89.2%
ER +, HER 2 +	34	5.8%
ER -, HER 2 -	21	3.6%
ER -, HER 2 +	8	1.4%

Comparing typical and atypical ILC, there was no difference in mean age at presentation (p = 0.4818). Typical ILC showed a tendency towards smaller tumour size, although this was not statistically significant (27.6 mm vs 32.2 mm (p = 0.081)). Eight patients had bilateral tumours, all of which were typical ILCs. The majority of typical ILCs were grade 2 (85.9% n = 500) and 352 (60.5%) were lymph node negative on final pathological staging. Atypical ILCs were more likely to be of higher histological grade; 35% were grade 3 compared to 9.6% of typical ILCs (p < 0.001). There was no statistical difference in the rate of LVI seen in typical and atypical ILCs; 15.6% and 23.3% respectively (p = 0.1797). Similarly, no statistically significant difference was observed in the rate of lymph node positivity; 199 (38.3%) vs 30 (47.6%) (p = 0.215), or in the mean number of positive lymph nodes; 5.93 vs 8.23 (p = 0.1884) in the two groups (Table 2).

**Table 2:** Comparison of Typical vs. Atypical ILCs.

	Typical (n = 519)	Atypical (n = 63)	*p-value*
**Mean age at presentation (years)**	60	61	0.4818
**Mean tumour size (mm)** N = 582	32.2	27.6	0.081
**Histological grade**			
**Grade 1**	9 (1.7%)	1 (1.6%)	0.009
**Grade 2**	460 (88.6%)	39 (61.9%)	<0.001
**Grade 3**	50 (9.6%)	22 (35.5%)	<0.001
**LVI (present)** n = 566	79 (15.6%)	15 (23.3%)	0.1797
**Nodal status** **Node positive** n = 578	199 (38.3%)	30 (47.6%)	0.215
**Mean no of positive lymph nodes**	5.93	8.23	0.1884

### 3.2 Response to neoadjuvant chemotherapy (NACT)

A total of 56 patients received NACT representing 6.9% of typical ILCs and 31.7% of atypical ILCs. pCR was observed in 3 (15%) patients with atypical ILC compared with none of the patients with typical ILC who received NACT. Two of the three patients with atypical ILC, in whom pCR was observed, had ER- HER2+ tumours and the third had a triple negative tumour. Overall comparison of RCB scores for both groups revealed a lower mean RCB score in patients with atypical ILCs (2.46) compared to those with typical ILCs (3.41) (p = 0.0365) (Table 3). Table 4 provides demographic and pathological information on those patients who received NACT. There was no statistically significant differences in the demographics and pathological features of those who received chemotherapy in the typical or atypical groups.

**Table 3:** Neoadjuvant chemotherapy.

	Typical (n = 519)	Atypical (n = 63)	*p-value*
Received NACT	36 (6.9%)	20 (31.7%)	<0.001
Did not receive NACT	483 (93.1%)	43 (68.3%)	<0.001
**Response**			
pCR	0 (0%)	3 (15%)	0.017
Patients with RCB Scores	23 (63.9%)	15 (75%)	
Mean RCB Score	3.41	2.46	0.0365

**Table 4:** Demographics of patients who received NACT.

	Typical (n = 36)	Atypical(n = 20)	*p-value*
**Mean age at presentation (years)**	51	54	0.2571
**Mean tumour size (mm)**	48.0	33.3	0.1903
**Histological grade**			
**Grade 1**	2 (5.6%)	1 (5%)	1
**Grade 2**	31 (86.1%)	15 (75%)	0.4989
**Grade 3**	3 (8.3%)	4 (20%)	0.3991
**LVI (present)**	13 (36.1%)	3 (15%)	0.1641
**Nodal status** **Node positive**	28 (77.78%)	13 (65%)	0.4717
**Mean no of positive lymph nodes**	8.69	4.6	0.1715

### 3.3 Recurrence free survival outcomes

**Fig. 1:**
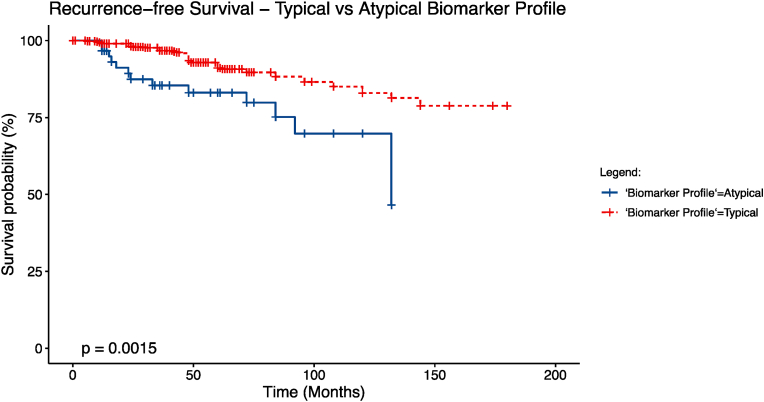
Kaplan Meier recurrence-free survival analysis for typical and atypical biomarker profiles.

Recurrence Free Survival (RFS) analysis revealed a five-year RFS of 91% for typical ILC and 83% for atypical ILC (p = 0.001) (Fig. 1). Median follow-up time was 72 months (Range 1–180 months).

Univariate analysis of clinicopathologic parameters revealed that patients with LVI were at increased risk of both local and distant disease recurrence, hazards ratio (HR) 2.82 (CI 1.67–4.76, p < 0.001). Additionally, patients with larger tumours, T3-4, HR 6.98 (CI 3.43-14.21 p < 0.001), N2, HR 6.90 (CI 3.41-13.99 p < 0.001), N3 HR 9.19 (CI 4.77-17.71 p < 0.001) or Nx lymph node status (lymph nodes were not or could not be sampled) HR 31.98 (CI 4.21-242.91 p < 0.001) had an increased risk of recurrence. Having completed NACT resulted in reduced recurrence-free survival in both typical, HR 3.99 (CI 1.92-8.29 p < 0.001) and atypical ILC subgroups, HR 6.51 (2.73-15.51 p < 0.001). There was an increased risk of recurrence in patients with ER-, HER2- tumours, HR 6.27 (CI 3.07-12.80 p < 0.001) (Table 5). No cases of disease recurrence were seen in patients with grade 1 tumours yielding infinite hazard ratios.

**Table 5:** Univariate analysis for recurrence free survival.

	HR (95% CI)	*p-value*
**Tumour Grade**		
Grade 1	1.00	
Grade 2	Inf	
Grade 3	Inf	
**LVI**		
Absent	1.00	
Present	2.82 (1.67–4.76)	<0.001
**NACT**		
Typical - Did not complete NACT	1.00	
Typical - Completed NACT	3.99 (1.92–8.29)	<0.001
Atypical – Did not complete NACT	1.85 (0.78–4.36)	0.162
Atypical – Completed NACT	6.51 (2.73–15.51)	<0.001
**Tumour Size**		
T1	1.00	
T2	2.57 (1.31–5.06)	0.006
T3-4	6.98 (3.43–14.21)	<0.001
**Nodal Stage**		
N0	1.00	
N1	1.42 (0.68–2.99)	0.351
N2	6.90 (3.41–13.99)	<0.001
N3	9.19 (4.77–17.71)	<0.001
Nx	31.98 (4.21–242.91)	<0.001
**Receptor status (biomarker profile)**		
ER + HER 2 – (Typical)	1.00	
ER +, HER 2 +	0.74 (0.18–3.05)	0.677
ER -, HER 2 -	6.27 (3.07–12.80)	<0.001
ER -, HER 2 +	1.35 (0.19–9.84)	0.764

On multivariate analysis, the correlation between reduced recurrence free survival and tumour size T2, T3-4, nodal positivity and ER-, HER2-biomarker profile remained statistically significant (Table 6). The increased risk of local and distant disease recurrence in these groups is independently calculated as HR 2.93 (CI 1.43-5.98 p = 0.003) for tumour size designated T2, HR 4.24 (CI 1.78-10.11 p < 0.001) for T3-4, HR 4.47 (CI 1.92-10.38 p < 0.001) and HR 5.8 (CI 2.53-13.31 p < 0.001) for N stages 2 and 3 respectively and HR 3.7 (CI 1.14-12.05 p = 0.03) for subtype ER-, HER2-.

**Table 6:** Multivariate analysis for recurrence free survival.

	HR (95% CI)	*p-value*
**LVI**		
Present	1 (1–1)	0.03
**NACT**		
Typical - Completed NACT	1.6 (0.64–4.02)	0.317
Atypical – Completed NACT	2.79 (0.8–9.67)	0.107
**Tumour Size**		
T2	2.93 (1.43–5.98)	0.003
T3-4	4.24 (1.78–10.11)	<0.001
**Nodal Stage**		
N2	4.47 (1.92–10.38)	<0.001
N3	5.8 (2.53–13.31)	<0.001
Nx	3.31 (0.37–29.89)	0.287
**Hormone Receptors**		
ER -, HER 2 -	3.7 (1.14–12.05)	0.03

R^2^ = 0.239.

While univariate analysis had revealed a statistically significant correlation between the presence of LVI, nodal stage Nx and having received NACT with increased risk of recurrence, these associations were not retained on multivariate analysis (Table 6).

## 4 Discussion

The unique clinical and pathologic features of ILC are well characterised, with emphasis on the need for distinguishing this breast cancer subtype from IBC-NST for prognostication, treatment selection and research [5]. Most notably, when comparing ILC with IBC-NST, the former shows an infiltrative pattern of dissemination, likely due to the loss of surface E-cadherin via CDH1 gene deletion or mutation [21]. ILC is the second most commonly observed tumour in patients with CDH1 germline mutations, a mutation more commonly associated with hereditary diffuse gastric cancers [22]. The metastatic pattern of ILC also contrasts with that of IBC-NST. IBC-NST shows a predilection for lung, liver, bone and brain while ILC classically spreads to the gastrointestinal tract, ovaries, peritoneum, retroperitoneum and leptomeninges [4,5,8,[23], [24], [25], [26]].

With recent advances in our understanding of breast tumour biology, it has become apparent that ILC is also a heterogeneous disease process. The majority of ILCs display classical morphology with a typical ER + HER2- biomarker profile [8,10], as observed in 89.2% of tumours in this retrospective cohort study of 582 patients. A minority of ILCs are characterised by an atypical biomarker profile (HER2+ or triple negative), more commonly seen in grade 3 tumours with pleomorphic or solid morphology. In our subgroup of atypical ILCs, grade 3 tumours were three times more frequent than in the typical ILC subgroup (35.5% vs 9.6% respectively). The overall rate of grade 3 tumours at 11.0% is consistent with the results of previous studies [27].

Larger tumour size (T2, T3-4) and N2 and N3 nodal stages were associated independently with poorer prognosis, with higher hazard ratio on both univariate and multivariate analysis (Table 5, Table 6). This is in keeping with the well-characterised behaviour of malignant disease and reflects the finding of preciously cited studies investigating lobular carcinoma [4,7,25].

Historically, NACT has been associated with suboptimal results in patients with typical ILC. Recording the presence or absence of pCR is commonly used to report response to NACT as pCR is a surrogate marker for increased recurrence-free survival [28]. A recent meta-analysis, including 87,303 patients, compared response rates to NACT in IBC-NST and ILC cohorts. While the authors reported increased pooled pCR rates in IBC-NST compared with ILC (22.1% v 7.4%, p=<0.001), results were not presented according to biomarker profile and many of the included studies did not report receptor status [14]. Analysis of the 56 patients who received NACT in the current study revealed that those with atypical ILC were more likely to achieve PCR (15% in atypical ILC versus 0% in typical ILC, p = 0.017). The overall pCR rate in our study was 5.36%, which is in keeping with the findings of other published cohorts [28,29]. The rate of pCR however was lower in atypical cases than has previously been reported [12], which might be related to the limited number of atypical cases in our study. However, this finding suggests that atypical ILC may actually achieve a pCR, unlike typical ILC.

RCB score post-NACT has been shown to be clinically useful with excellent reproducibility and proven long-term prognostic significance in all subtypes of breast cancer [30]. It provides an additional tool outside of the binary reporting of pCR for comparing response to NACT [6]. RCB scores are categorised into four classes, from RCB 0 (pCR), through RCB-I, RCB-II and RCB-III in order of increasing residual disease burden. In their study, designed to validate the prognostic utility of RCB scores in different molecular subtypes of breast cancer, Yau et al. reported a disproportionately low number of patients with ILC. This was considered likely to be due to the perception that patients with ILC do not respond well to neoadjuvant chemotherapy and are therefore not selected for neoadjuvant treatment [6]. Our study shows an improved response to NACT in patients with atypical ILC compared to typical ILC both in terms of pCR and mean RCB (2.46 vs 3.41, p = 0.0365). This provides a rationale for further investigation into the potential for a greater role for the administration of NACT to patients with atypical ILC.

In ER + HER2-breast cancers, the Oncotype DX 21 gene assay has been developed and validated for determining the risk of recurrence and potential benefit of adjuvant chemotherapy for an individual patient [31,32]. The same issues arise from pooling ILC and IDC in studies evaluating the clinical usefulness of this risk assessment tool and there are conflicting reports on the validity of the 21-gene assay in patients with ILC [33]. Likely due to the perception that ILC is less responsive to chemotherapy, patients with high recurrence scores are less commonly treated with adjuvant chemotherapy when compared with equivalent IDC [[34], [35], [36]]. There is as yet no similar tool for evaluating the potential benefit of NACT in patients with ILC, or indeed that of adjuvant chemotherapy in ER-tumours.

Regarding survival outcomes in our cohort, patients who received NACT had a worse recurrence-free survival compared to those who did not receive NACT (atypical HR 6.51 CI 2.73-15.51 p < 0.001, typical HR 3.99 CI 1.92-8.29 p < 0.001). This is likely due to the fact that patients who were selected for NACT had high-risk and biologically aggressive disease. Furthermore, this historical cohort precedes the CREATE X and KATHERINE trials which have enabled a second opportunity to treat non-responding patients using adjuvant therapies such as T-DM1 and Capecitabine [37,38].

Our study has a number of limitations. All data were retrospectively evaluated and some information, including family history and menopausal status at the time of diagnosis, was not recorded for all patients. Although the overall study patient cohort is of considerable size, only 10.8% (63/582) of tumours displayed atypical tumour biomarker profile in keeping with the published literature. Additionally, the use of any adjuvant therapy was not recorded or included in analyses. This single-centre study included also patients from 2005 when adjuvant therapy regimes differed from those currently available such that the overall study findings may not be entirely representative of contemporary medicine.

### 4.1 Conclusions

Notwithstanding the above limitations, the results of this study demonstrate the importance of recognising the heterogeneity of ILC in which clinical behaviour and response to therapy is influenced by the biomarker profile of the tumour. Tumour size (T2+) and lymph node status (N2-3) are independent predictors of reduced recurrence-free survival in ILC, irrespective of treatment regime. Patients with typical and atypical ILC biomarker profiles differ in their response to NACT, with better responses seen in patients exhibiting atypical biomarker profiles.

## CRediT authorship contribution statement

**N.P. Quirke:** Writing – review & editing, Writing – original draft, Project administration, Investigation, Formal analysis, Data curation. **C. Cullinane:** Writing – review & editing, Writing – original draft, Supervision, Project administration, Methodology, Investigation, Formal analysis, Data curation, Conceptualization. **M.A. Turk:** Writing – review & editing, Formal analysis. **N. Shafique:** Formal analysis, Data curation. **D. Evoy:** Writing – review & editing, Supervision. **J. Geraghty:** Writing – review & editing, Investigation. **D. McCartan:** Writing – review & editing, Project administration, Methodology, Investigation, Data curation, Conceptualization. **C. Quinn:** Writing – review & editing, Writing – original draft, Supervision, Methodology, Investigation, Formal analysis, Conceptualization. **J.M. Walshe:** Writing – review & editing, Methodology, Investigation, Conceptualization. **E. McDermott:** Supervision, Methodology, Investigation, Conceptualization. **C. Rutherford:** Writing – review & editing, Supervision, Methodology, Investigation, Formal analysis, Conceptualization. **R.S. Prichard:** Writing – review & editing, Writing – original draft, Supervision, Project administration, Methodology, Investigation, Formal analysis, Data curation, Conceptualization.
